# Phytochelators Intended for Clinical Use in Iron Overload, Other Diseases of Iron Imbalance and Free Radical Pathology

**DOI:** 10.3390/molecules201119725

**Published:** 2015-11-23

**Authors:** Christina N. Kontoghiorghe, Annita Kolnagou, George J. Kontoghiorghes

**Affiliations:** Postgraduate Research Institute of Science, Technology, Environment and Medicine, 3 Ammochostou Street, Limassol 3021, Cyprus; xtina_jt@hotmail.com (C.N.K.); koranita@cytanet.com.cy (A.K.)

**Keywords:** phytochelators, iron overload, iron metabolism, metals, antioxidants, therapeutics, thalassaemia, cancer, longevity

## Abstract

Iron chelating drugs are primarily and widely used in the treatment of transfusional iron overload in thalassaemia and similar conditions. Recent *in vivo* and clinical studies have also shown that chelators, and in particular deferiprone, can be used effectively in many conditions involving free radical damage and pathology including neurodegenerative, renal, hepatic, cardiac conditions and cancer. Many classes of phytochelators (Greek: phyto (φυτό)—plant, chele (χηλή)—claw of the crab) with differing chelating properties, including plant polyphenols resembling chelating drugs, can be developed for clinical use. The phytochelators mimosine and tropolone have been identified to be orally active and effective in animal models for the treatment of iron overload and maltol for the treatment of iron deficiency anaemia. Many critical parameters are required for the development of phytochelators for clinical use including the characterization of the therapeutic targets, ADMET, identification of the therapeutic index and risk/benefit assessment by comparison to existing therapies. Phytochelators can be developed and used as main, alternative or adjuvant therapies including combination therapies with synthetic chelators for synergistic and or complimentary therapeutic effects. The development of phytochelators is a challenging area for the introduction of new pharmaceuticals which can be used in many diseases and also in ageing. The commercial and other considerations for such development have great advantages in comparison to synthetic drugs and could also benefit millions of patients in developing countries.

## 1. Introduction

Many plant products and almost all classes of plant polyphenols found mainly in fruit and vegetables are promoted for healthy dietary reasons and considered as potent antioxidants with beneficial effects in the health of normal individuals [1]. Many such natural compounds or related active ingredients of plant origin are also sold over the counter due to their described therapeutic benefits in almost all diseases and ageing, although in many cases these therapeutic claims are anecdotal or unconfirmed. No therapeutic antioxidant is prescribed in clinical practice [2].

In theory, a plant product antioxidant, including dietary antioxidants for human use, is a substance derived from plants which prevents, quenches, inhibits or decreases the toxic effects arising from free radicals, reactive oxygen species, reactive nitrogen species, as well as their toxic intermediates and other related by-products which can cause damaging effects to tissues and normal physiological functioning in humans [3,4,5,6]. Since free radicals and the other toxic by-product intermediates such as lipid peroxides are also related to ageing and associate tissue damage, the use of antioxidants is linked to longevity. In all cases of antioxidant action specific conditions and selective antioxidant activity related to a particular toxicity pathway are required [2].

Despite the commercial success, the therapeutic use of plant products in common clinical practice is not yet established due to many reasons, such as the lack of efficacy and toxicity data which are required for regulatory approval by the health authorities. Major stumbling blocks in the application of therapeutic plant products is the cost of development and the competition due to commercial interests from established big pharmaceutical companies which have monopolies and lucrative profits from competing synthetic patented drugs [7].

The number of clinical trials in different diseases with plant therapeutic products is increasing at present in order to receive regulatory approval and also confirm their therapeutic value and claims despite their use in traditional folk medicine for hundreds of years in different countries worldwide [8]. Their application is particularly important in diseases where no established treatment is available or effective and also in developing countries where the cost of synthetic drugs is not affordable [7].

Among many of the plant products with bioactive properties, there are hundreds of plant derived compounds with metal binding and chelating properties. The plant products with chelating properties, namely phytochelators (Greek: phyto (φυτό)—plant, chele (χηλή)—claw of the crab) should have at least two metal binding sites which can form complexes with a metal ion as the closing member of a ring structure. Phytochelators can compete with other natural or synthetic chelators for metal ions and can affect the metal metabolic properties and associated biochemical or pharmacological pathways in humans and other organisms.

The mode of action of such plant derived compounds with metal binding properties is governed by the same physicochemical, thermodynamic and kinetic parameters as those described for other chelators *in vitro* [9,10]. Similarly, their mode of action *in vivo* depends on many other physiological and pharmacological parameters such as the level of their absorption, distribution, metabolism, excretion and toxicity (ADMET) and also the extent of interactions involving other drugs and many biomolecules including proteins of the metal metabolic pathways [2,11,12].

**Figure 1:**
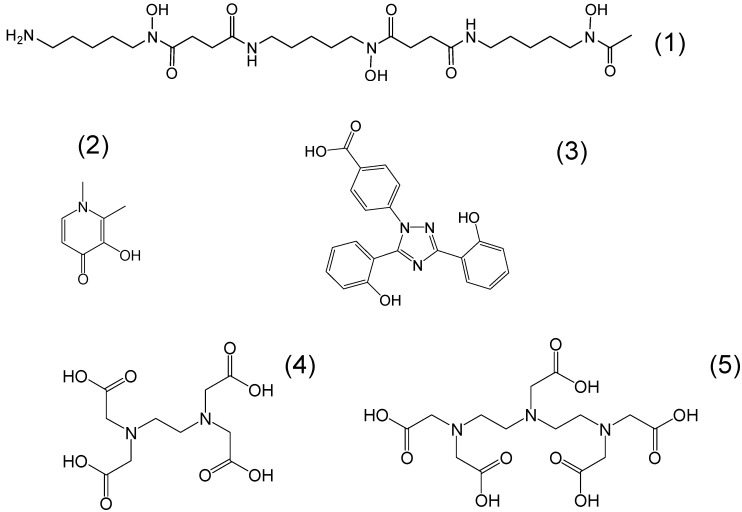
The chemical structure of the main iron chelating drugs. The main iron chelating drugs which are commercially available for the treatment of transfusional iron overload are deferoxamine or *N*′-{5-[acetyl(hydroxy) amino]pentyl}-*N*-[5-({4-[(5-aminopentyl)(hydroxy)amino]-4-oxobutanoyl}-amino)pentyl]-*N*-hydroxysuccinamide (**1**), deferiprone or 3-hydroxy-1,2-dimethylpyridin-4(1*H*)-one (**2**) and deferasirox or 4-[(3*Z*,5*E*)-3,5-bis(6-oxo-1-cyclohexa-2,4-dienylidene)-1,2,4-triazolidin-1-yl]-benzoic acid (**3**). The other two chelating drugs EDTA or 2-({2-[bis(carboxymethyl)-amino]ethyl}(carboxymethyl)amino)acetic acid (**4**) and DTPA or 2-[bis[2-[bis(carboxymethyl)amino]-ethyl]amino]acetic acid (**5**) are not currently used for iron detoxification but for other metal detoxification and other clinical conditions (see text).

One of the most common groups of diseases related to metal metabolic imbalance are iron metabolism abnormalities. Iron is an essential metal required for the normal growth and development of all organisms including plants and animals. It is estimated that a quarter of the human population suffers from iron deficiency anaemia at some stage of their life [13]. There are many other abnormalities related to iron metabolism, including iron overload from increased iron absorption and from regular red blood cell transfusions (Table 1) [9,10]. One of the diseases with the highest morbidity and mortality rate related to iron metabolism is thalassaemia, which if not treated with regular transfusions is lethal within the first few years of life [14,15]. In countries where transfusions are available thalassaemia patients die from cardiac iron overload toxicity by the age of 20, unless chelation therapy is introduced [14,15]. It is estimated that 100,000 babies are born with thalassaemia every year worldwide and most die without treatment [7,14]. The iron chelating drugs used in the treatment of thalassaemia are deferoxamine (DF), deferiprone (L1) and deferasirox (DFRA) (Figure 1). Deferiprone and DFRA are orally active whereas DF is not orally active and administered subcutaneously or intravenously. Deferiprone is a bidentate (two metal binding sites) chelator forming a 3L1:1Fe complex, DFRA is a tridentate (three metal binding sites) chelator forming a 2DFRA:1Fe complex and DF is a hexadentate (six metal binding sites) chelator forming a 1DF:1Fe complex at physiological pH [10]. The therapeutic and other properties of the chelating drugs in relation to thalassaemia including efficacy, toxicity and cost have been previously reviewed [7,10]. Other synthetic chelators widely used are ethylenediaminetetraacetic acid (EDTA) and diethylenetriaminepentaacetic acid (DTPA). The former is used as a multipotent drug by millions of patients in alternative medicine and the latter for the detoxification of toxic metals such as plutonium (Figure 1) [16].

Iron chelating drugs intended for the treatment of iron overload in thalassaemia or other similar conditions should be able to compete with endogenous iron proteins such as transferrin and ferritin and to remove intracellular excess stored iron causing its excretion [10]. A chelating drug is effective if it causes an increase in iron excretion at a rate higher than the rate of iron intake from transfusions and iron absorption.

In contrast to chelators intended for the treatment of iron overload, iron chelating drugs intended for the treatment of iron deficiency anaemia should be able to increase iron absorption and increase haemoglobin production to normal physiological levels. Lipophilic chelatos, such as 8-hydroxyquinoline (or quinolin-8-ol) have previously been shown to increase iron absorption and the deposition of excess iron in tissues [17]. Chelators can also be used in the treatment of the anaemia of chronic disease and other abnormalities of iron metabolism by redistributing iron and achieving normal physiological levels in the tissues (Table 1) [18,19].

Phytochelators and synthetic chelators could also be used in many other therapeutic applications by targeting a wide spectrum of diseases related to free radical toxicity and pathology as well as ageing (Table 1) [20]. In such cases sufficient therapeutic concentrations should be able to reach the targeted tissue(s) without causing toxic side effects. For example chelators intended for the treatment of neurodegenerative diseases such as Alzheimer’s and Parkinson’s diseases should be able to cross the blood brain barrier in addition to the other pharmacological and therapeutic effects.

**Table 1:** Prospects of targeting therapies of iron phytochelators in iron overload and other clinical conditions.

**Iron Overload**
Haemoglobinopathies: β-thalassaemia major, β-thalassaemia intermedia, HbE β-thalassaemia, HbS β-thalassaemia, sickle cell anaemia
Anaemias: Aplastic anaemia, sideroblastic anaemia, Blackfan-Diamond anaemia, Fanconis anaemia, pernicious anaemias, congenital dyserythropoietic anaemia, hereditary hypochromic anaemia
Hereditary conditions: Idiopathic haemochromatosis, hereditary spherocytosis, pyruvate-kinase deficiency, congenital atransferrinaemia, porphyria cutanea tarda
Iatrogenic: Intramuscular iron dextran, dietary or iatrogenic iron intake, iron poisoning
Other conditions: Haemolytic disease of the newborn, iron overload in liver disease, iron overload in haemodialysis
**Iron Imbalance and Oxidative Stress**
Friedreich’s ataxia, Hallevorden-Spatz syndrome, Parkinson’s disease, Alzheimer’s disease
Cyclooxygenase and lipoxygenase inhibitors
Congestive cardiac failure, liver disease, acute kidney disease, rheumatoid arthritis
Ischaemia reperfusion injury
Drug toxicity, e.g., doxorubicin induced cardiac damage
**Iron Imbalance**
Anaemia of chronic disease in inflammatory, infectious and neoplasmic diseases
Iron deficiency anaemia.
**Free Radical Pathology**
All diseases affected by free radical damage and oxidative stress
Ageing
**Metal Toxicity, Diagnostics and Therapeutics**
Aluminium overload
Actinide contamination, e.g., plutonium and uranium
Diagnostic metal complexes, e.g., gallium, indium and gadolinium
Therapeutic metal complexes, e.g., gold and platinum
**Other Metal Imbalance and Toxicity Conditions**
All cancer types with increased iron requirements, neoplasmic disease, neuroblastoma, hepatocellular carcinoma (Adjuvant therapies with anticancer drugs)
**Infectious Diseases**
All microbial infections, e.g., meningitis, malaria and other parasitic infections, mucormycosis. (Adjuvant therapies with antimicrobial drugs)

Adapted from reference [9].

In principle, effective phytochelators could have many therapeutic applications similar to the existing synthetic chelating drugs. In this context phytochelators could be used as main, alternative or adjuvant therapy in many conditions including combination therapies with synthetic chelators for synergistic and or complimentary effects (Table 1) [9,10,20].

## 2. Molecular Aspects of Iron Chelation Therapy

Chelators are organic compounds which possess at least two metal binding sites with electron donor atoms such as N, O and S, which have affinity for metal ions and the appropriate proximity between them to form a ring with the metal ion as the closing member. Within this context there are many organic biological molecules, including plant compounds possessing electron donor atoms, which can be involved in metal complex formation. The donor atoms can be present in the metal binding sites of acidic groups such as -OH, -COOH, -SH, -NOH, where the proton could be displaced by the metal ion or in Lewis bases such as -C=O, -NH_2_, -O-R, -OH, -S-R.

There are many classes of iron chelators including microbial siderophores, phytochelators and chelating drugs, as well as many drugs and biomolecules such as proteins, fatty acids, sugars, ATP, DNA and RNA with chelating potential for iron [5,9,10,21,22,23]. The general aspects of iron chelation and the prospects for the development of iron chelators intended for clinical use have been previously reviewed [9,10]. Many chemical parameters can influence the metal binding properties of a chelator such as the type and stereospecific arrangement of the metal binding groups, the electron releasing or withdrawing and steric effects of other substituents in the chelator backbone structure, and the number of metal binding sites involved in metal coordination [9,10].

The functional groups with chelating potential usually have variable affinity for several metal ions. The metal stability constants of L1 for example are in the following order Fe > Cu > Al > Zn [24]. Similarly, transferrin which is a specific protein for chelating and transporting iron in plasma, has binding sites with high affinity for iron but also lower affinity for many other metal ions such as Al, In and Ga [25].

In the case of each chelator, the metal complex or complexes being formed have different physicochemical, pharmacological and toxicological properties in comparison to the chelator or the metal ion involved in the chelator metal complex [9,10].

Physicochemical parameters such as water solubility, lipid/water partition, stability in atmospheric air and sunlight are important parameters for the selection of chelators for further development. The biochemical, pharmacological and toxicological properties of chelators, their metabolites and metal ion complexes are also among the most important parameters which can determine the prospects for the selection of chelators for further development and clinical use.

In principle, iron chelating drugs have to compete with transferrin, lactoferrin, ferritin and other endogenous naturally occurring chelators for iron at all the sites and stages of absorption, metabolism and excretion of the chelating drugs and their metal ion complexes. The competition between transferrin with chelating drugs for iron, as well as other interactions including the interaction between the chelating drugs for iron and other metal ions is governed by thermodynamic and kinetic parameters [24,25]. Deferiprone, for example, can be involved in iron removal or donation with transferrin under certain conditions, which are mainly dependent on the concentration and the saturation of L1 and transferrin with iron [26]. However, iron exchange between transferrin and DF is very slow and of no pharmacological significance in iron overload due to kinetic restrictions imposed by the chemical structure of DF and its iron complex. Similar forms of interactions have also been shown between competing chelators such as L1 and DF and their iron complexes, as well as other drugs which have iron binding properties [24].

There are different methods of assessment of the affinity of chelators for iron and other metals. One such method is the determination of the metal stability constants (log β) which in the case of the iron chelating drugs, L1 appears to have the highest stability constant for iron (log β = 35) by comparison to DF (log β = 31) and DFRA (log β = 27) (Table 2) [10]. EDTA and DTPA are less specific for iron and during their clinical use in iron loaded patients in addition to an increase in iron excretion, the excretion of zinc, copper and magnesium also appear to increase [27]. Minor increases in zinc excretion were also rarely observed in a few iron loaded patients receiving intravenous DF or oral L1 [28,29]. In relation to the other metal affinities, L1 and DF can be used for the removal of aluminium from aluminium loaded patients in addition to iron removal [30].

**Table 2:** Physicochemical properties of phytochelators and iron chelating drugs.

Chelator	log β	MWt	Kpar	Kpar Iron Complex	Charge
Deferoxamine	31	561	0.02	0.02	positive
Deferiprone	35	139	0.18	0.05	neutral
Deferasirox	27	373	6.30	-	negative
Maltol	30	126	1.23	0.32	neutral
Tropolone	32	122	3.04	4.50	neutral
Mimosine	36	198	0.01	0.01	zwitterionic

Iron(III) stability constants (log β); molecular weight (MWt); *n*-octanol/water partition coefficients (Kpar) of chelator and chelator iron complex (Kpar Iron complex); charge of chelator and chelator iron(III) complex at physiological pH (Charge). Adapted from reference [10].

In general the *in vitro* estimations of the affinity of chelating drugs for iron and other metal ions, such as the estimation of stability constants or other physicochemical parameters cannot reflect the ability of the chelating drugs to remove iron *in vivo*. In particular, the toxicity, pharmacological, pharmacokinetic and metabolic properties of a chelating drug may limit its ability to bind and remove sufficient amounts of various toxic iron forms which may be present *in vivo* [9,10]. Many other factors may affect the efficacy of chelating drugs *in vivo* including the number, bioavailability and toxicity of metabolites and their chelating properties. Similarly, other parameters such as the lipid/water partition and rate of clearance of their iron complexes, may also influence the overall efficacy of the chelating drug in iron removal and also their toxicity (Table 2) [9,10].

## 3. The Role and Effect of Low Molecular Weight Iron Chelators, Including Phytochelators, on Iron Metabolism and Toxicity

There are many naturally occurring low molecular weight organic molecules including phytochelators and other plant products, as well as synthetic organic molecules like medicinal drugs which possess metal ion binding groups and can form iron and other metal complexes. The role and effect of these organic metal binding molecules on iron and other metal metabolism, as well as free radical toxicity implications is not yet fully investigated or characterised. The low molecular weight naturally occurring chelators found in cells, e.g., ATP, ADP, citrate or some of which are absorbed from food, e.g., ascorbate and polyphenols, as well as drugs and other molecules containing iron binding groups can all be considered to be involved in the formation of an intracellular “transit” low molecular weight iron pool [5,23,26,31]. A “transit” low molecular weight iron pool is also thought to be present in mitochondria and other subcellular organelles and operate using similar mechanisms [32].

Some of the components of this “transit” low molecular weight iron pool form iron complexes which affect iron metabolic pathways, including the exchange of their iron with apo-proteins and its transfer to the iron domains of proteins (Table 3) [10,31].

**Table 3:** Examples of iron-containing proteins and their function, which could be influenced or targeted by phytochelators.

Protein	Function
Haemoglobin	Oxygen transport
Myoglobin	Oxygen transport
Cytochromes	Electron transport. Respiration
Adrenodoxin	Electron transport. Oxidation/reduction
Ferredoxin	Electron transport. Oxidation/reduction.
Cyt P450 and b5	Drug detoxification
Ribonucleotide reductase	DNA synthesis
Proline hydroxylase	Collagen synthesis
Peroxidases	Decomposition of hydroperoxides
Catalase	Decomposition of hydronen peroxide
Lipoxygenase	HPETE and leukotriene synthesis
Cyclooxygenase	Prostaglandin and thromboxane synthesis
Aconitase	Tricarboxylic acid cycle
Succinate dehydrogenase	Tricarboxylic acid cycle
NADH dehydrogenase	Electron transport. Respiration
Xanthine oxidase	Conversion of xanthine to uric acid
Aldehyde oxidase	Metabolism of aldehydes
Transferrin	Iron transport in plasma
Lactoferrin	Iron binding in milk and other secretions
Ferritin	Iron storage
Haemosiderin	Iron storage
Hephaestin	Protein affecting iron metabolism
Ferroportin	Protein affecting iron metabolism
Hepcidin	Protein affecting iron metabolism

Adapted from reference [25].

It is anticipated that the iron uptake and release processes that are involved intracellularly to and from the naturally occurring chelators and their iron complexes are governed by the same thermodynamic and kinetic parameters as for other chelators and their metal complexes [33]. For example citrate (10 mM, in plasma) and glutathione in cells (5 mM, in liver cells), as well as other low molecular weight naturally occurring chelators may form low molecular weight iron complexes intracellularly and in plasma, similar but to a much lesser extent in comparison to the iron chelating drugs L1 and DF [20].

Under iron overload conditions a low molecular weight iron pool of non-transferrin bound iron (NTBI) is formed in plasma when usually transferrin is fully saturated with iron [26,34]. This form of iron and also of iron found bound to natural chelators which can be present in plasma and intracellularly can potentially facilitate the catalytic formation of toxic free radicals and oxidative stress damage [26,34].

Plant products with metal binding properties and especially phytochelators, which can increase the size of the low molecular weight iron pool, may play a role in the urinary or faecal elimination and overall excretion of iron similar to the mode of action of the chelating drugs L1, DF, and DFRA [33]. In contrast, the mode of action of lipophilic phytochelators may resemble that of the lipophilic chelator 8-hydroxyquinoline, which decrease the size of the low molecular weight iron pool by diverting iron or depositing iron to tissues and overall may minimise iron excretion and have the opposite effect to L1 and DF and other hydrophilic chelators, *i.e.*, lipophilic iron chelators may increase the body iron load and also intracellular free radical toxicity [17]. Usually the clinical efficacy and toxicity effects of phytochelators and other low molecular weight iron chelators depends on the level of effective concentrations that can achieve *in vivo* for maximum pharmacological activity without the cause of serious toxicity.

The mode of action of phytochelators and other naturally occurring chelating molecules can have variable effects on iron metabolism and free radical toxicity, similar to those observed during the use of the chelating drugs L1, DF and DFRA [2,10]. For example, unlike DFRA, both L1 and DF appear to decrease iron absorption both in animals and humans and can be used in acute iron poisoning [35,36,37,38,39]. Similar effects have been observed during iron absorption studies with metal binding drugs such as tetracycline [40]. In contrast, other compounds such as the lipophilic chelators maltol, 8-hydroxyquinoline, omadine (or 1-hydroxypyridine-2-thione) and 2-hydroxy-4-methoxy-pyridine-1-oxide (L6) have been shown to increase iron absorption [17,35].

There are a large number of iron chelators of microbial and plant origin, which have variable affinity for iron and can play a major role in iron absorption and excretion, as well as have pro-oxidant or antioxidant effects, through their iron or copper complexes. Metal ion binding organic compounds and phytochelators, including some which may be present in food, have not yet been fully investigated for their iron, copper and other metal binding properties. Almost all plant antioxidants including polyphenols, caffeic acid, ascorbic acid *etc.* have iron binding affinities and can affect iron metabolism, as well as the catalytic activity of iron in the formation of free radicals [23,41,42]. Ascorbic acid for example can cause reduction of ferric to ferrous iron, which can result in an increase of iron absorption from the gut but also under certain conditions it can also cause an increase in the free radical production and damage caused by iron catalysis [43]. In addition, combination of ascorbic acid with DF is widely used for increasing iron excretion in iron loaded thalassaemia patients [44].

Some examples of powerful iron chelators of plant origin which were studied more systematically in relation to iron metabolism and toxicity are mimosine, tropolone and maltol, which are known to affect iron metabolic pathways and also to inhibit free radical formation (Table 2) [41].

In general phytochelators like in the case of chelating drugs have different iron chelating properties and can be used in a variety of iron metabolic pathways and diseases of iron metabolic imbalance and iron related free radical pathology (Table 1, Table 2 and Table 3). There are also prospects for the use of phytochelators in combination therapies with synthetic chelators and also with other antioxidants for synergistic and or complimentary effects [9,10].

## 4. The Role of Free Radicals and Antioxidants in Biological Systems

The essential role of biologically formed free radicals and related oxygen and nitrogen activated products found in many biological systems and pathways have been previously reviewed [2,3,4,5,6,20]. In normal physiological conditions the formation of free radicals and other reactive oxygen and nitrogen species such as the hydroxyl radical, superoxide, nitrogen oxide, hydrogen peroxide and lipid peroxides is primarily dependent on iron and copper catalytic centers involved in redox reactions [2,3,4,5,6,20].

**Figure 2:**
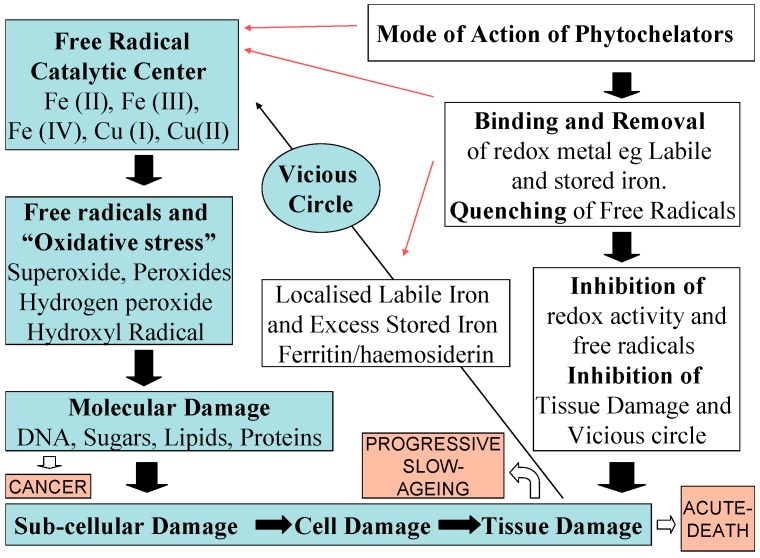
The role of phytochelators in inhibiting free radical formation and damage. Vicious circle of free radical toxicity catalysed by iron and copper catalytic centres lead to oxidative stress and molecular, subcellular, cellular and tissue damage and associated diseases such as cancer, acute iron toxicity and ageing.

In general, free radicals and other reactive oxygen species such as hydrogen peroxide are involved in many biological processes including the oxidation of food products, the metabolism of natural products, drugs and other xenobiotic molecules, the destruction of invading microbes through phagocytosis, the destruction of senescent and other cells and of cellular components *etc.* [2,20]. However, under certain conditions free radicals and other reactive oxygen species can also cause modification or damage to virtually all known organic biomolecules found in cells including DNA, sugars, proteins and lipids (Figure 2) [3,4,5,6].

In biological systems the formation of free radicals is in most cases initiated by iron and copper catalytic centres present in proteins and to a smaller extent by low molecular weight complexes of these two metals. The rate of production of free radicals under normal conditions is well regulated and controlled by specific metabolic antioxidant pathways and redox buffering antioxidant systems such as those provided by oxidized/reduced glutathione, as well as by dietary or other antioxidant molecules [20,45].

Under free radical metabolic imbalance conditions, excess free radical production and oxidative stress can cause acceleration in the damage of organic biomolecules such as lipid peroxidation and subsequent damage to cells, tissues and organs. The same free radical mechanisms can cause initiation of diseases such as cancer by causing DNA damage and repeated mutations, acceleration of ageing and in some less common cases, e.g., in acute iron poisoning, total irreversible organ damage and death (Figure 2) [2,20]. In the latter case the body’s antioxidant processes collapse and the cellular oxidation processes are accelerated and cannot be controlled or the associated damage be repaired, leading to the modification and destruction of organic biomolecules and irreversible damage to cells, tissues and organs [2,20].

One of the major classes of antidotes to oxidative stress damage is the antioxidants. There are many publications describing the properties, role and mode of action of many antioxidants, mainly of plant origin. There is also wide publicity and advertisements suggesting that antioxidants in food, beverages, skin creams, *etc.*, are important components needed for healthy living and longevity. Many nutraceuticals, mainly of plant origin, are also sold over the counter as potent antioxidants, which are mostly promoted as food supplements for healthier living. Within this context many natural molecules, mainly of plant origin, as well as many synthetic molecules have been shown to have antioxidant activity using both *in vitro* and *in vivo* models [2,20,46]. Despite that under normal conditions dietary antioxidants may be sufficient for offering protection against free radical toxicity, in clinical conditions there is not as yet any specific or effective antioxidant treatment prescribed for any disease related to free radical toxicity and pathology [2,20,46].

The introduction of pharmaceutical antioxidants has many limiting factors not only related to clinical application and targeting in clinical conditions but also related to the cost of commercial development. Among the major drawbacks in the introduction of antioxidant therapy are the lack of antioxidant specificity, tissue targeting and possibility of toxicity. Factors related to the pharmacological application include the dose and timing of administration of the antioxidant, the possibility of interference with other treatments related to the underlying condition and also other effects associated with the duration of administration such as the possibilities for prophylactic, long and short term uses [2].

In considering the specificity and targeting aspects of antioxidants there are many pathways which are involved in the generation, proliferation and distribution of free radicals, reactive oxygen and nitrogen species that could cause molecular, cellular, tissue and organ damage. Examples of causes of free radical and oxidative stress damage include UV light, ionising radiation, heavy metals, environmental pollution, iron and copper overload, mitochondrial malfunction and dietary imbalance [2]. In all these and many other cases several forms of intervention including the use of specific antioxidant will be needed for targeting, preventing or inhibiting the damaging effects.

The widely known mode of action of most classical antioxidants such as the vitamins A, C and E and polyphenols in general, is the quenching of free radicals and other related oxygen and nitrogen reactive species. However, phytochelators with iron and copper binding sites may have a more effective antioxidant activity by preventing the catalytic formation of free radicals and free radical cascades through inhibition of the redox catalytic centers involving iron and copper [2,20,41,47,48]. In principle, iron and copper chelating drugs including phytochelators can prevent free radical damage by binding these two metal ions and rendering them redox inactive. Furthermore, effective polyphenolic phytochelators specific for iron and copper can prevent, as well as quench free radicals and related toxicity.

## 5. Antioxidant Targeting Aspects of Iron Phytochelators and Chelating Drugs

There are many pharmacological and other limitations and considerations for the prospect of introducing targeted therapeutic antioxidants in different clinical conditions. Despite many encouraging *in vitro* results, the introduction of targeted antioxidant therapies in clinical conditions is based on the ability of the antioxidant to reach therapeutically effective concentrations and have low toxicity in the affected tissues of the clinical condition(s) [4,43]. In addition, several other aspects of therapeutic targeting and application in a specific clinical condition can be considered, which is similar to other drug selection such as the risk/benefit assessment of the new therapy by comparison to existing therapies, pharmacological properties which may affect the efficacy of the antioxidant such as absorption, distribution, metabolism, elimination and toxicity (ADMET), ability to reach specific organs such as the brain or the heart, interaction with other drugs, costs, *etc.* [2,7,12,20,48].

In each clinical condition the mode of antioxidant activity and the molecular pathway, cellular compartment, tissue and organ targeted is different for each antioxidant and depends on its chemical and pharmacological properties. Within this context and considering the mode of action at the molecular level, the basis of the antioxidant properties of phytochelators such as polyphenols with chelating properties is their ability to inhibit the redox catalytic activity of iron and copper in the formation of free radical cascades, as well as the quenching of the excess free radicals which are continuously formed during oxidative stress. Accordingly, several critical parameters are required in each case of antioxidant therapeutic targeting, such as the timing of intervention, the duration of administration and the selection of the appropriate dose for achieving maximum therapeutic effect [2,7,12,20,48].

Many forms of interactions of phytochelators and other chelators could affect iron and copper catalysed free radical production and related oxidative stress damage, such as inhibition or modification of the relevant enzymatic activity or other molecular metal complex oxidant activity. The interactions of phytochelators with iron or copper containing enzymes and other molecular metal ion complexes could result in many different effects such as metal removal, donation and exchange, ternary complex formation between the chelator and the protein through the metal, allosteric interactions of a side chain of the protein with the chelator or metal chelator complex, redox changes and the catalytic oxidation/reduction of biomolecules [2,20].

In general, chelators can directly influence redox changes initiated by iron and copper catalytic centers or indirectly by influencing the turnover and metabolic pathways of these metals. An example of a potent antioxidant system operating in plasma in relation to iron is the mode of action of the plasma chelating protein transferrin, which removes toxic low molecule weight iron and also oxidizes ferrous to ferric iron, forming ferric but not ferrous iron complexes [25,26]. A similar mechanism of potential prevention of free radical damage catalysed by iron can also take place in the presence of the iron chelating protein lactoferrin, which is found in bodily secretions and in neutrophils [49].

Similar mode of antioxidant action to transferrin and lactoferrin is displayed by effective iron chelators and chelating drugs. Within this context the chelating drugs L1, DF and other chelators with similar iron binding sites convert aqueous ferrous iron into ferric iron and form ferric iron complexes within seconds at physiological pH [26,50]. Furthermore, under physiological conditions the iron complexes of L1 and DF are redox inert, especially when the chelators are present in excess over iron. This is particularly important in the treatment of iron overload where the concentration of chelating drugs is sufficiently high for inhibiting free radical catalytically active low molecular weight iron complexes, provided the administration of the chelating drugs is continuous and the doses are sufficiently effective for reaching therapeutic concentrations.

Iron chelators such as L1 and DF can inhibit free radical toxicity through other pathways of redox changes including the reduction of tetravalent iron(IV) in haem into ferric iron [51]. In general haem containing tetravalent iron(IV) is considered as a more toxic species and source of redox catalytic iron involved in the formation of toxic free radicals by comparison to low molecular weight iron(II and III). Similarly, chelating agents can interact with iron containing proteins in a different way, for example DF can increase the rate of oxidation of haemoglobin to methaemoglobin at physiological pH or 2,3-dihydroxybenzoic acid can cause oxidation of iron in iron-containing proteins, for example of cytochrome C [52,53]. In a different experimental model it has been shown that the iron and copper complexes of plant phytochelator products containing the catechol moiety and also synthetic chelators such as L1 can act as superoxide radical scavengers [54].

In each experimental model the inhibitory and other interaction effects by chelators on free radical formation and related free radical damage are concentration and rate dependent, suggesting that bioavailability and achievement of optimal therapeutic concentrations are major limiting factors in antioxidant activity and targeting [2,8,11,12].

The antioxidant targeting efficacy of chelators depend on many parameters which are related to their chemical, biochemical, pharmacological and other properties. Within this context different selection criteria apply for chelators targeting the iron or copper catalysed free radical toxicity and related damage which are usually initiated in different intracellular compartments or extracellularly.

In all cases the targets of chelating drugs in iron or non-iron loading conditions are mostly stored iron, low molecular weight iron complexes or intracellular iron containing enzymes. For example, chelators have different accessibility to intracellular compartments and only chelators that can cross the cell membrane can have direct access to intracellular iron containing enzymes involved in free radical reactions and cascades such as cyclooxygenase and lipoxygenase [2] (Table 3).

The turnover of intracellular iron containing enzymes can also be affected indirectly through other biological pathways. For example, the inhibition of the turnover of an iron containing enzyme can be accomplished by the mobilisation of intracellular iron, which is in the low molecular weight transit iron pool and which is utilised for incorporation into the apoproteins. The turnover of these enzymes can also be mediated through chelator interactions with the iron transporting or regulatory proteins such as transferrin and hepcidin, which are directly related to the turnover of the intracellular transit iron pools. Furthermore, a different pathway of targeting with similar effects can be mediated through interactions with the receptors of these and other iron transporting and regulatory proteins. Overall, there are many iron containing enzymes and related metabolic pathways, which in each case could be targeted or affected by phytochelators and other chelators (Table 3) [9].

Other interactions such as allosteric effects of chelators with the iron containing enzymes and other proteins may also influence their free radical and iron metabolic activity. For example, in circular dichroism studies L1 was reported to induce a conformational change in haemoglobin and the reduction of the helicity of the protein to depend on the concentrations of L1 [55].

The various forms of interactions of phytochelators and other chelators with iron and also copper could in general be used to target free radical toxicity arising from the catalytic centers containing these metals. In each case specific targeting of iron and copper containing enzymes and other proteins, as well as storage compartments of these metals and also of low molecular weight transit metal pools may be used for new strategies in the design of new pharmaceutical antioxidants for a variety of conditions including cancer, inflammatory and neurodegenerative diseases and also ageing.

## 6. Phytochelators with Iron Binding Properties

There are hundreds of plant products with metal binding or chelating sites and many with reported antioxidant properties. Although the antioxidant effects of these compounds are widely described in the literature, the metal binding effects are not so well studied and documented. Although this field covers a large area of biomedicine most of the antioxidant mechanisms in relation to iron have not yet been fully explored. Similarly, in most cases the iron binding effects of phytochelators have not been studied and developed with aim of their further development for clinical application. Of particular interest are the iron binding phytochelators which can play important therapeutic role both as antioxidants and as iron chelating agents in many diseases related to iron metabolism, similar to those reported for L1 and the other chelating drugs (Table 1) [2,20].

An empirical approach in the identification of potent phytochelators is their resemblance of the iron binding site to the iron chelating drugs L1, DF, DFRA, EDTA and the microbial siderophores. The major chelating sites of microbial siderophores are mainly the catechol moiety, which is found in bacteria and the hydroxamate moiety which is found in fungi [21]. There are also many other chelating site variations in different microbial species involving mainly O, N and S as electron donor atoms in bidentate, tridentate and hexadentate composition and also as part of linear or ring structures, with aromatic, heteroaromatic or non-aromatic backbone configuration [56]. Other side chain substituents which are not involved in the metal binding site are also important in a chelator structure and affinity for metal ions. These substituents may cause electronic, steric, solubility, variation in the lipid/water partition, as well as many other effects, all of which can influence the pharmacological and toxicological properties of the chelator. It should be noted that DF is of fungal origin (*Streptomyces pilosus*) and L1 was designed to mimic the α-ketohydroxy metal binding site of the orally active phytochelators mimosine, tropolone and maltol which have high affinity for iron (Table 2) [57,58,59].

The characteristics of each natural or synthetic iron chelator and the properties required for possible clinical application in many different conditions have been reviewed and highlighted in the previous sections [9,10]. Of major interest and priority is the identification of effective and safe phytochelators, which can be used clinically in many categories of transfusional iron loaded patients who are not currently adequately treated due to the toxic side effects or unaffordable high cost of chelating drugs [7,14]. Similarly, the identification of phytochelators which can increase iron absorption and be used in the treatment of iron deficiency anaemia, can also benefit millions of patients worldwide [13,33]. In contrast, phytochelators which can prevent iron absorption may have a use in the treatment of hereditary haemochromatosis, acute iron poisoning and the prevention of colorectal cancer [9,10,38,60].

Many other possible clinical applications of phytochelators such as the treatment of the anaemia of chronic disease, infections, detoxification of other metals *etc.* could also be envisaged similar to previous findings with other chelators and iron chelating drugs (Table 1) [18,19,25,61,62].

The bifunctional activity of specific phytochelators with iron removal and antioxidant quenching effects may have a use in neurodegenerative diseases and many other clinical conditions of tissue damage as previously shown mainly with L1 and also other chelating drugs [32].

Another possible application of phytochelators is their development as combination therapies with existing drugs. In all cases of clinical application of combination therapies sufficient evidence of therapeutic improvement and safety is required in comparison to monotherapies and also evidence that the combination has significant advantages over existing therapies.

Overall, the possible clinical application of phytochelators is based on many factors which are also applicable to other drugs such as the risk/benefit assessment in comparison to existing therapies, the cost and availability of the phytochelators to patients, the drug regulatory authorities permits and restrictions for use, the influence of competing pharmaceutical companies, marketing issues and many others [7].

### Chemistry of Phytochelators

Hundreds of phytochelators with different metal chelating sites can be identified from the chemical structure of many plant products. The chelating site characteristics, as well as the backbone and side chain characteristics of each phytochelator can influence their biological, pharmacological, toxicological and other effects. Within this context some of the general chemical characteristics of a number of phytochelators can be described to illustrate their ability to influence iron metabolic pathways and also to be considered for further development and application in clinical conditions related to iron metabolism and free radical pathology.

**Figure 3:**
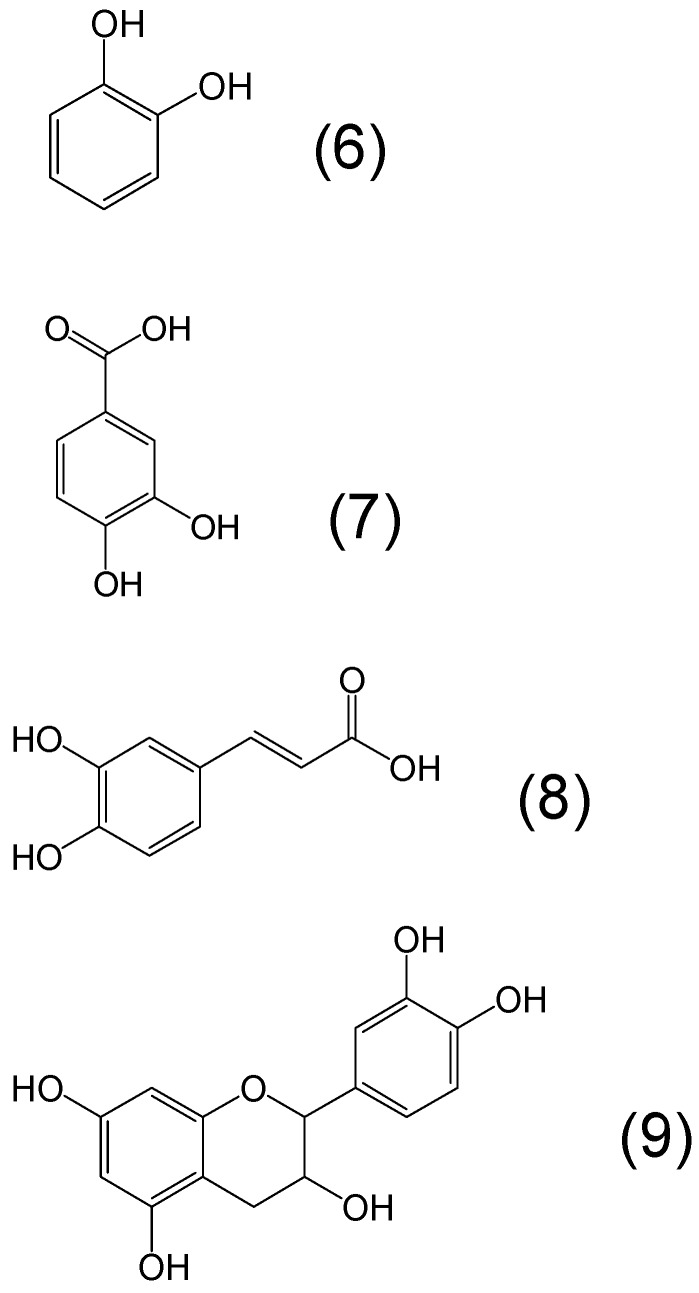
Examples of phytochelators with the chemical structure containing catechol as the metal binding site moiety. Catechol or benzene-1,2-diol (**6**), protocatechuic acid or 3,4-dihydroxybenzoic acid (**7**), caffeic acid or 3-(3,4-dihydroxyphenyl)-2-propenoic acid (**8**) and catechin or 2-(3,4-dihydroxyphenyl)-3,4-dihydro-2*H*-chromene-3,5,7-triol (**9**).

The most common phytochelators with wide distribution in plant products including fruits and vegetables are the polyphenols which contain a catechol moiety metal chelating site and have many biological and antioxidant properties (Figure 3) [63,64,65,66,67]. Characteristic examples of catechol moiety- containing phytochelators are caffeic acid, protocatechuic acid and catechin. The latter is a flavan containing catechol as a member of a tricyclic ring structure and can be found in dimeric or polymeric forms containing more catechol rings. Although catechol forms iron complexes with high stability constants, the iron complex is not stable at physiological pH [57]. Carboxylic acid and other substituents on the catechol ring may increase stability, e.g., protocatechuic acid and other dihydroxybenzoic acids (Figure 3).

**Figure 4:**
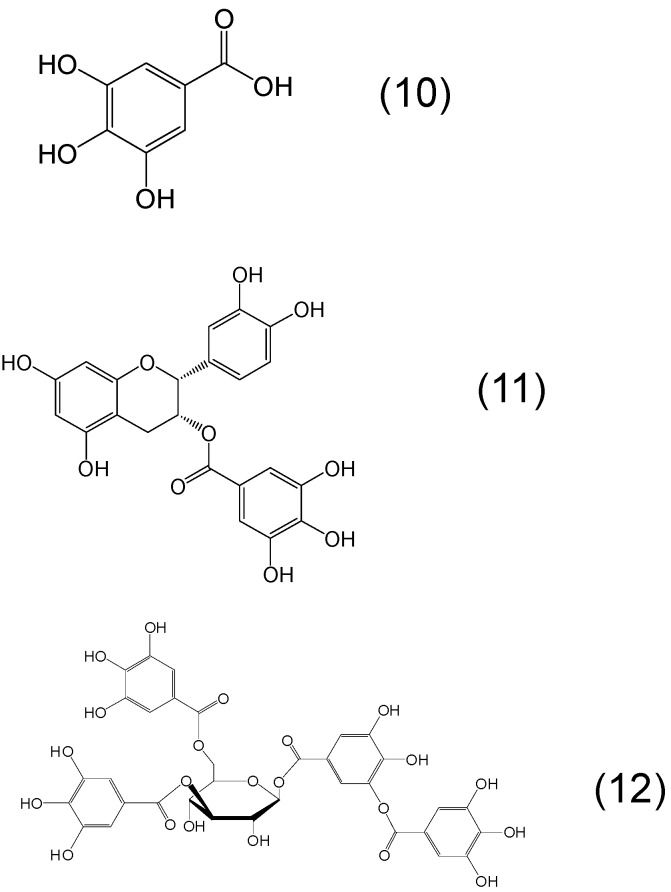
Examples of phytochelators with the chemical structure containing trihydroxybenzene as the metal binding site moiety. Gallic acid or 3,4,5-trihydroxybenzoic acid (**10**), epigallocatechin or (2*R*,3*R*)-2-(3,4,5-trihydroxyphenyl)-3,4-dihydro-2*H*-chromene-3,5,7-triol (**11**) and tannic acid or 2,3-dihydroxy-5-({[(2*R*,3*R*,4*S*,5*R*,6*R*)-3,4,5,6-tetrakis({3,4-dihydroxy-5-[(3,4,5-trihydroxyphenyl)carbonyloxy]phenyl}carbonyloxy)oxan-2-yl] methoxy}carbonyl)phenyl 3,4,5-trihydroxybenzoate (**12**).

Another common polyphenol class of chelators with the trihydroxybenzene iron binding site is the gallic acid containing phytochelators (Figure 4). Stereometrically it is likely that only two of the adjacent hydroxyl groups are involved in iron binding, similar to catechol. Combination of catechol and gallate chelating sites both with iron binding affinity within the same molecule can also be identified in some polyphenols, e.g., epicatechin gallate and in different tannin molecules (Figure 4).

**Figure 5:**
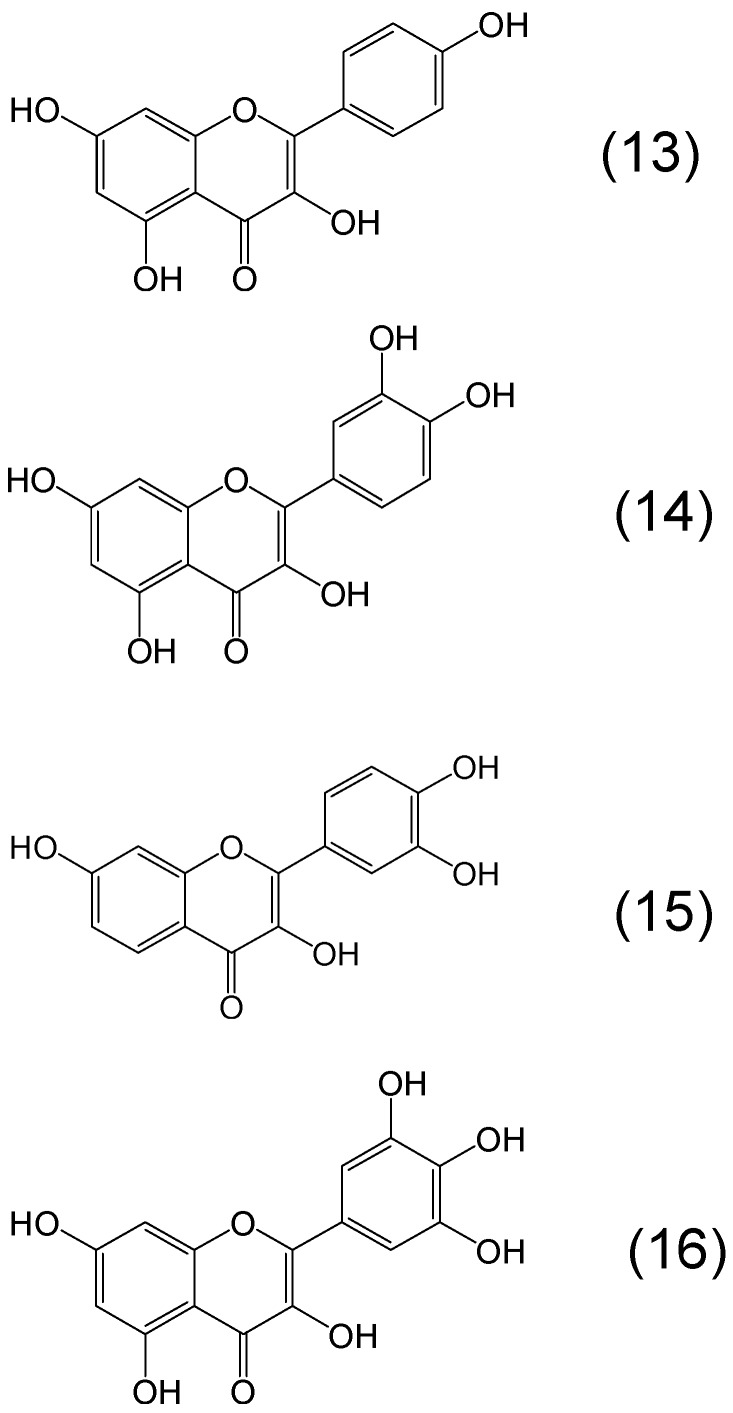
Examples of phytochelators with the flavonoid chemical structure. Kaempferol or 3,5,7-trihydroxy-2-(4-hydroxyphenyl)-4*H*-chromen-4-one (**13**), quercetin or 2-(3,4-dihydroxyphenyl)-3,5,7-trihydroxy-4*H*-chromen-4-one (**14**), fisetin or 2-(3,4-dihydroxyphenyl)-3,7-dihydroxychromen-4-one (**15**), myricetin or 3,5,7-trihydroxy-2-(3,4,5-trihydroxyphenyl)-4-chromenone (**16**).

Flavonoids are a class of polyphenols with different iron binding sites (Figure 5). For example in kaempferol both an α-ketohydroxy group in the C ring and a β-ketohydroxy one between the A and C rings are potential binding sites for iron. Similarly, quercetin contains the iron binding sites of kaempferol but also contains a catechol moiety metal binding site in the B ring, whereas myricetin contains the iron binding sites of kaempferol and a gallate instead of the catechol iron binding site in the B ring. Another biologically important flavonol with two iron binding sites, one catechol in the B ring and another alpha ketohydroxy binding site in the C ring is fisetin (Figure 5) [68].

**Figure 6:**
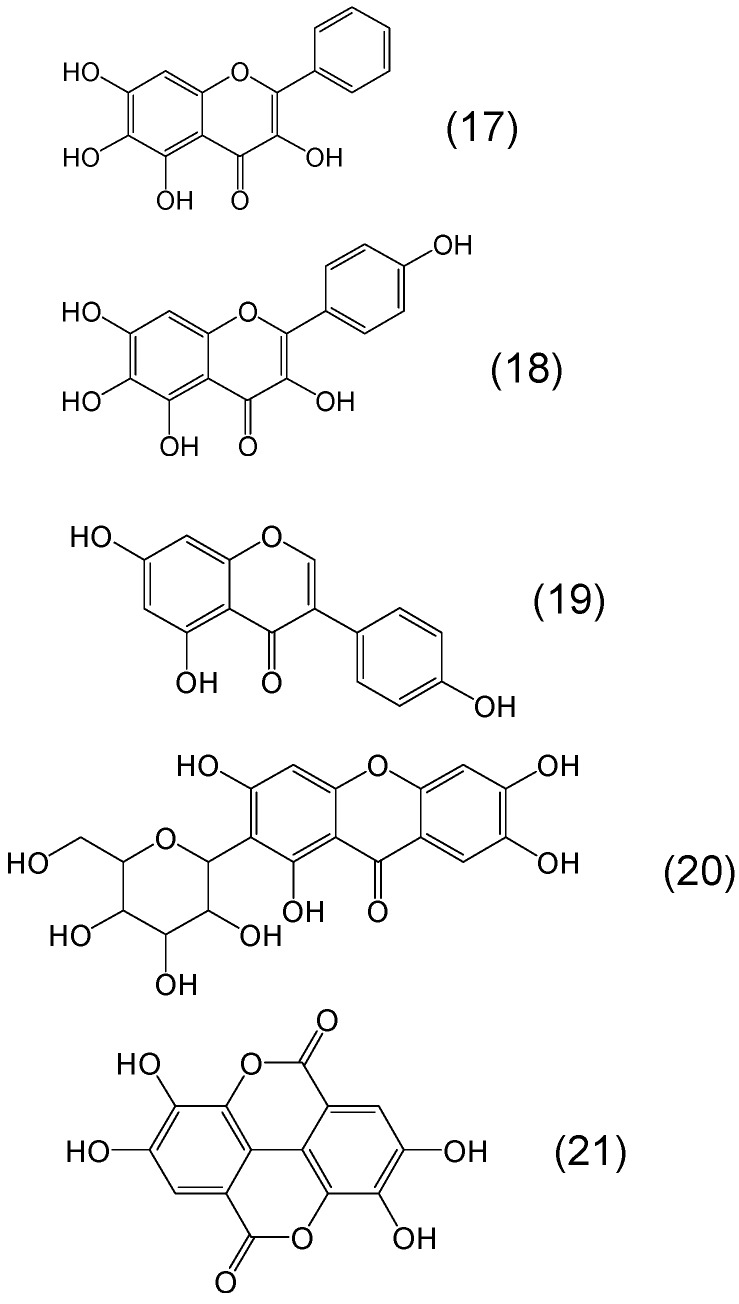
Examples of phytochelators with the flavonoid, isoflavonoid and xanthonoid chemical structure. Baicalein or 5,6,7-trihydroxy-2-phenyl-chromen-4-one (**17**), scutellarein or 5,6,7,4′-tetrahydroxyflavone (**18**), genistein or 5,7-dihydroxy-3-(4-hydroxyphenyl)chromen-4-one (**19**), mangiferin or (1*S*)-1,5-anhydro-1-(1,3,6,7-tetrahydroxy-9-oxo-9*H*-xanthen-2-yl)-d-glucitol (**20**), and ellagic acid or 2,3,7,8-tetrahydroxy-chromeno[5,4,3-cde]chromene-5,10-dione (**21**).

There are many other classes of phytochelator polyphenols containing either catechol or gallate or both moieties in different positions on an aromatic or semiaromatic ring structure with high potential for iron binding. Examples of this category are the flavones baicalein and scutellarein or the isoflavones genistein and mangiferin and also ellagic acid (Figure 6).

In general all catechol and gallate moiety-containing polyphenols have iron, copper and other metal binding properties, as well as free radical quenching and other antioxidant or prooxidant properties [69,70].

**Figure 7:**
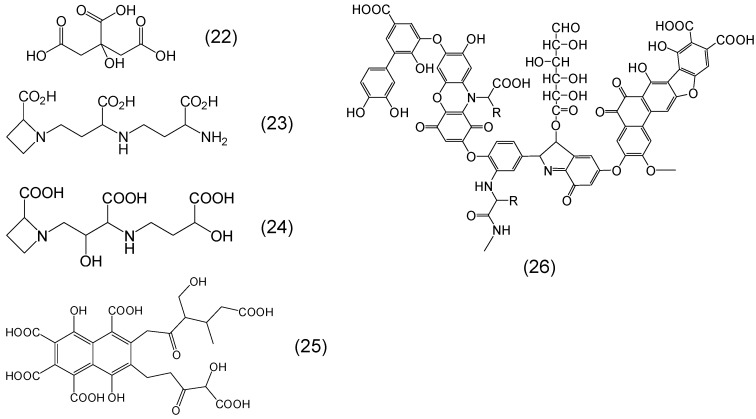
Examples of phytochelators containing the carboxylic acid as the metal binding site. Citric acid or 2-hydroxypropane-1,2,3-trioic acid (**22**), nicotianamine or *N*-(*N*-(3-amino-3-carboxypropyl)-3-amino-3-carboxypropyl)azetidine-2-carboxylic acid (**23**), mugineic acid or (2*S*)-1-[(2*S*,3*S*)-3-carboxy-3-[[(3*S*)-3-carboxy-3-hydroxypropyl]amino]-2-hydroxypropyl]azetidine-2-carboxylic acid (**24**), fulvic acid or 3,7,8-trihydroxy-3-methyl-10-oxo-1,4-dihydropyrano[4,3-*b*]chromene-9-carboxylic acid (**25**) and humic acid (**26**).

In addition to polyphenolic phytochelators there are many other phytochelators with different chelating sites. For example, derivatives containing carboxylic acid metal ion binding sites are present in all plants and are involved in iron chelation. Among these are citrate, nicotianamine, mugineic acid, fulvic acid and humic acids (Figure 7) [71,72,73,74,75]. A typical humic acid has a variety of components including quinone, phenol, catechol and sugar moieties.

**Figure 8:**
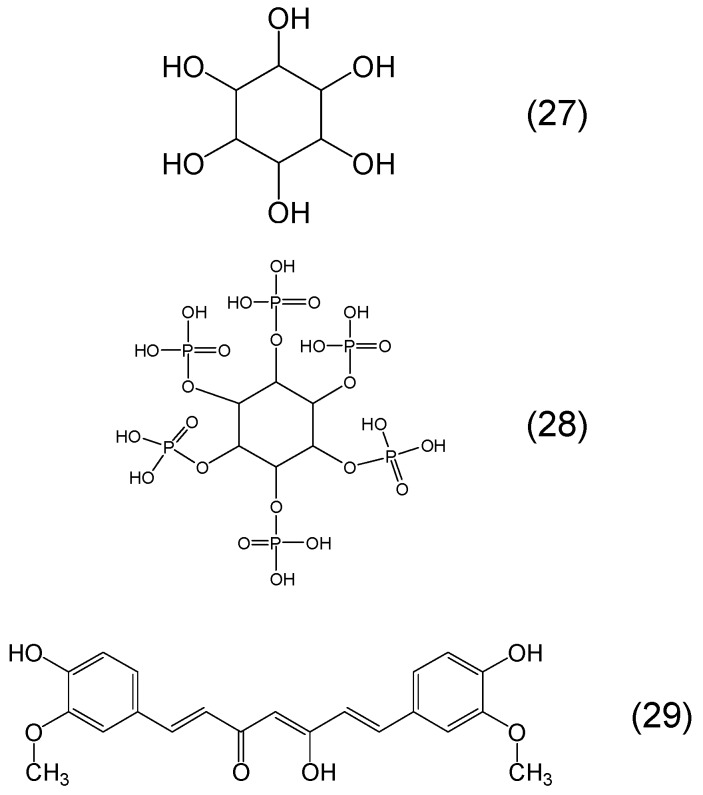
Examples of phytochelators containing hydroxyl or phosphate as the metal binding site. Inositol or cyclohexane-1,2,3,4,5,6-hexol (**27**), inositol hexaphosphate (phytic acid) or (2,3,4,5,6-pentaphosphonooxycyclohexyl) dihydrogen phosphate (**28**) curcumin or (1*E*,6*E*)-1,7-bis(4-hydroxy-3-methoxyphenyl)hepta-1,6-diene-3,5-dione (**29**).

Sugars including mono-, di-, and polysaccharides and other plant products such as inositol with adjacent dihydroxy groups can also bind iron, but have much weaker affinity for iron than for example polyphenols containing the catechol moiety [76]. Curcumin is a weak chelator containing a beta keto enol metal binding site on a linear chain configuration (Figure 8) [77]. Another large group of plant products with high affinity binding sites for iron are the phosphate containing derivatives such as ATP, ADP, AMP and the inositol phosphates [5,78,79,80]. Inositol hexaphosphate (IP6) or phytic acid is a well-known phytochelator with a variety of biological activities including that of the principal storage form of phosphorus in many plant tissues, especially bran and seeds (Figure 8) [78,79,80]. Similar iron binding properties are shown by other inositol phosphates, which also exhibit many other biological activities.

**Figure 9:**
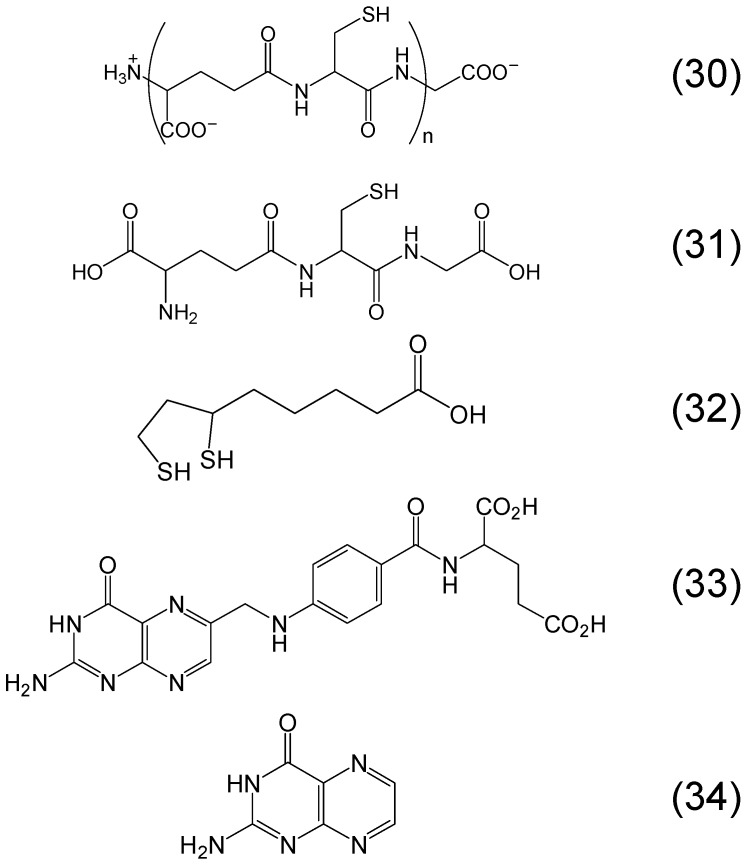
Examples of phytochelators containing a thiol or pterin in the metal binding site. Phytochelatin (*n* = 2–11) (**30**), glutathione or (2*S*)-2-amino-5-[[(2*R*)-1-(carboxymethylamino)-1-oxo-3-sulfanylpropan-2-yl]amino]-5-oxopentanoic acid (**31**), dihydrolipoic acid (6,8-dimercapto-octanoic acid) (**32**), folic acid or (2*S*)-2-[[4-[(2-amino-4-oxo-1*H*-pteridin-6-yl)methylamino]benzoyl]-amino]pentanedioic acid (**33**) and pterin or 2-amino-1*H*-pteridin-4-one (**34**).

Several other classes of phytochelators involving different metal binding sites can be identified in many other plant products. Phytochelatins is a large class of phytochelators which are glutathione oligomers, usually composed by 2–11 monomers and like glutathione involve a thiol (-SH) group in their metal binding sites. Among the functions of phytochelatins is the detoxification of heavy metals and antioxidant activity similar to glutathione (Figure 9) [20,81,82]. Another phytochelator with similar antioxidant and heavy metal detoxification properties to glutathione is dihydrolipoic acid (or 6,8-dimercaptooctanoic acid) which is the reduced form of lipoic acid and is found in plants in different quantities [82,83]. Dihydrolipoic acid contains two thiol (-SH) groups involved in its metal binding site (Figure 9) [82,83]. Other examples of phytochelators are folic acid and other pterin-containing compounds which have two potential metal binding sites, one involving the keto group and the adjacent N and the other the two adjacent N of the heterocyclic pteridine ring (Figure 9) [5,84].

**Figure 10:**
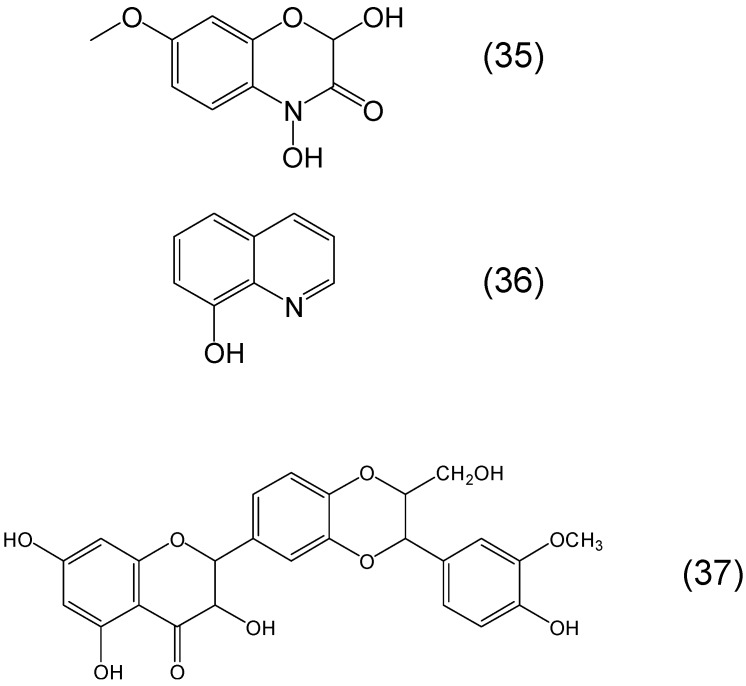
Examples of phytochelators containing a cyclic hydroxamate in the metal binding site and phytochelators used in clinical diagnostics and clinical trials. DIMBOA or 2,4-dihydroxy-7-methoxy-1,4-benzoxazin-3-one (**35**), 8-hydroxyquinoline or quinolin-8-ol (**36**), silibinin or (2*R*,3*R*)-3,5,7-trihydroxy-2-[(2*R*,3*R*)-3-(4-hydroxy-3-methoxyphenyl)-2-(hydroxymethyl)-2,3-dihydro-1,4-benzo-dioxin-6-yl]-2,3-dihydrochromen-4-one (**37**).

The production of hydroxamates in plants is rare, unlike microbial siderophores where hydroxamates are widely produced. However, some cyclic hydroxamates such as DIMBOA (2,4-dihydroxy-7-methoxy-1,4-benzoxazin-3-one) have been identified which may have potential for iron and other metal binding both through the cyclic hydroxamate or the α-ketohydroxy metal binding site (Figure 10) [85,86].

**Figure 11:**
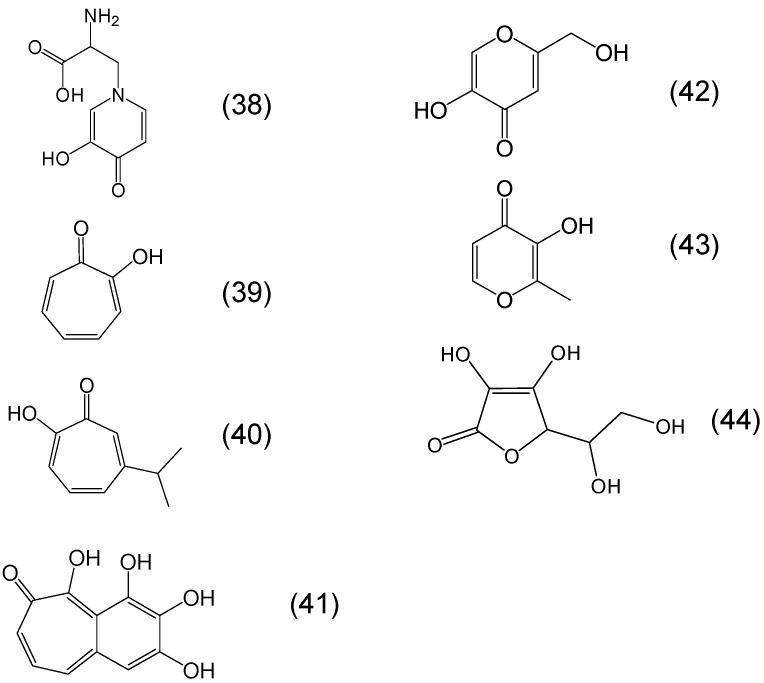
Examples of phytochelators containing the alpha ketohydroxy group in the metal binding site. Mimosine or 2-amino-3-(3-hydroxy-4-oxopyridin-1-yl)propanoic acid (**38**), tropolone or 2-hydroxycyclohepta-2,4,6-trien-1-one (**39**), hinokitiol or 2-hydroxy-6-propan-2-ylcyclohepta-2,4,6-trien-1-one (**40**), purpurogallin or 2,3,4,5-tetrahydroxybenzo[7]annulen-6-one (**41**), kojic acid or 5-hydroxy-2-(hydroxymethyl)pyran-4-one (**42**), maltol or 3-hydroxy-2-methylpyran-4-one (**43**), ascorbic acid or (2*R*)-2-[(1*S*)-1,2-dihydroxyethyl]-3,4-dihydroxy-2*H*-furan-5-one (**44**).

Ascorbic acid is another heterocyclic ring molecular structure containing an α-ketohydroxy as well as a dihydroxy metal binding site (Figure 11). Ascorbic acid is known to have various physiological effects involving both metal chelating and reducing properties, all of which are concentration dependent. The oxidised form of ascorbic acid, namely dehydroascorbic acid which has three oxo groups is a much weaker chelator than the reduced form of ascorbic acid [87,88].

A class of phytochelators with continuous biological and clinical interest for iron chelation is the α-ketohydroxy heteroaromatic group and other related chelators. The importance of this class of chelators was highlighted following the development of L1 for clinical use [57,58,59]. In particular, the iron chelating properties of the α-ketohydroxy binding site in three different backbone groups including the pyridine ring, e.g., mimosine, the pyrone ring, e.g., maltol and kojic acid, the cycloheptatriene ring, e.g., tropolone and the benzocycloheptene ring, e.g., purpurogallin were previously identified and studied for iron chelation and antioxidant activity (Figure 11) [41,57].

Overall many classes of phytochelators have been shown to fulfill the requirements for iron and other metal binding. The extent of the biological and pharmacological activity of each of the phytochelators in relation to iron metabolism depend on their affinity for iron and many other properties, which are affected by several parameters as previously described for other chelators intended for clinical use [9,10,57,59]. Similarly, phytochelators intended for the treatment of iron overload have to fulfill many other criteria and specific requirements, especially the ability to achieve negative iron balance in regularly transfused patients and not to be toxic at effective therapeutic doses. Different properties and requirements apply for chelators intended for clinical use in other diseases of iron metabolism such as iron deficiency anaemia, hereditary haemochromatosis, acute iron poisoning *etc.* [9,10,20,25,33].

## 7. Screening of Phytochelators Intended for Clinical Use in the Treatment of Iron Overload

There are increasing prospects for the development of phytotchelators for the treatment of iron overload and other diseases related to other iron metabolic disorders. In addition, the clinical application of phytotchelators can involve antioxidant treatments in many other diseases related to oxidative stress and tissue damage, ageing and other metal metabolic disorders.

No serious efforts have been undertaken so far for the systematic screening, selection and development of phytochelators for the treatment of iron overload in regularly transfused patients, mainly due to commercial reasons. However, there is an increasing need for such development especially in relation to thalassaemia patients in developing countries who cannot afford the cost of expensive imported chelating drugs [7]. The development of phytotchelators can also benefit many thalassaemia patients in developed countries who experience toxicity or non-efficacious treatment with the present chelating drugs.

In addition to iron overload, phytotchelators can have application in many other abnormalities of iron metabolism such as hereditary haemochromatosis, iron deficiency anaemia, anaemia of chronic disease and acute iron poisoning. Phytotchelators can also be used in other metal intoxications, e.g., copper and aluminium overload and many other diseases, similar to the application of L1 and other chelating drugs (Table 1) [9,10,20,25]. Further therapeutic benefits can also be obtained from the development of phytotchelators in combination with the chelating drugs and many other drugs targeting different therapeutic pathways including antioxidant therapies.

### 7.1. Initial Screening of Phytotchelators Intended for Clinical Use in Conditions of Iron Metabolism and Free Radical Pathology

Several phytotchelators have been originally identified and tested during the various stages of the design and screening of orally active chelators intended for the treatment of thalassaemia which led to the development of L1 [57,58,59]. The interest on the development of phytochelators for clinical use was later postponed because of insufficient commercial interest for their further screening and clinical development and also because the available resources were mainly used for the development of the leading iron chelator, namely L1 [7,57,58,59].

Within this context the phytotchelators maltol, catechol and 2,3-dihydroxybenzoic acid together with other synthetic chelators were used in the original preclinical screening system which involved *in vitro* iron chelation at different pH and other conditions and also protein, cell and animal studies [57]. In the initial screening of the phytochelators only maltol has shown some promising iron binding affinity results but no significant *in vivo* results of iron removal from iron-loaded mice in a model resembling the treatment of iron overload [57]. Subsequently, several other phytochelators were investigated in different experimental screening models of iron removal and antioxidant activity including mimosine, maltol, kojic acid, caffeic acid, pyrogallol, tropolone and purpurogallin. The screening programme was aimed at selecting phytochelators for clinical potential both as effective iron chelators and antioxidants. The same screening system was also used for identifying toxic effects both for the chelators and their iron complexes in relation to different metabolic pathways and also the possibility of promotion of oxidative stress toxicity [41].

In one of these screening procedures the effect of different concentrations (5–400 μM) of phytochelators and other chelators on the production of thiobarbituric acid reactive substances formed from damage to deoxyribose by iron(II) in the presence of hydrogen peroxide and ascorbic acid was examined. In general, all chelators including L1, mimosine, maltol, kojic acid, 2,3-dihydroxybenzoic acid and caffeic acid inhibited the breakdown of deoxyribose in a concentration dependent manner except purpurogallin and EDTA which appeared to activate this process [41].

In another screening procedure studying the effect of different chelator concentrations (5–100 μM) on the fluorescence of UV irradiated IgG, some synthetic chelators and kojic acid caused activation of the process, whereas all other phytochelators, including maltol, caused moderate inhibition [41]. Similarly, almost all the above phytochelators showed strong inhibitory effect of oxidative damage in a different screening system studying the effect of chelators (500 μM) on the production of thiobarbituric acid reactive substances by skeletal muscle homogenates. In this screening system catechol was the most effective inhibitor (97%), and kojic acid the least effective (67%) [41].

In a subsequent comparative screening study of eleven chelators intended for clinical use including four phytochelators namely maltol, kojic acid, 3,4-dihydroxybenzoic acid and mimosine the rate of iron mobilisation from iron-59 labeled ferritin and from hepatocytes labeled with iron-59 transferrin, as well as microsomal lipid peroxidation induced by Fe (II)/ADP and NADPH was investigated. The most effective chelators in iron mobilisation and of inhibition of lipid peroxidation were L1 and mimosine, whereas the other three phytochelators maltol, kojic acid and 3,4-dihydroxy-benzoic acid were less effective [89].

Further studies on the antioxidant effects of chelators in different *in vitro*, *in vivo* and clinical conditions were predominantly concentrated on L1, which became an approved chelating drug for the treatment of iron overload and was commercially available [2].

Many other experimental toxicity models have been used to identify other possible toxic side effects of phytochelators and synthetic chelators intended for clinical use such as *in vivo* and cells studies. The identification of different pathways of toxicity for experimental drugs in *in vitro* and *in vivo* studies is essential for diagnostic and prophylactic measures in clinical settings and also for patient safety [7].

In general the toxicity profile of each drug including phytochelators and the chelating drugs DF, L1 and DFRA is different and for example in the latter case the causes of the toxicity may not be related to iron or other metal chelating properties of the drug [9,10].

### 7.2. The Screening of Iron Phytochelators and Other Chelators in Cell Models of Iron Metabolism

Different *in vitro* and *in vivo* studies were carried out to identify the biological, toxicological and pharmacological properties of phytochelators and also of other chelators in a screening procedure involving the selection and identification of chelators intended for clinical use. Within this context the importance of parameters such as the lipid/water partition coefficient of the phytochelators and their iron complexes was compared to their ability to transfer iron across the red blood cell membrane in a number of incubation experiments using iron-59 labeled complexes for periods of 30–180 min [57,90]. In these experiments the neutral and lipophilic phytochelators maltol and tropolone caused an increase in iron incorporation in red blood cells at 48% and 78% respectively. In contrast, iron incorporation in red blood cells from the charged or hydrophilic phytochelators mimosine, kojic acid, catechol, caffeic acid, pyrogallol and 2,3-dihydroxybenzoic acid was much lower (3%–13%) [57,90]. Further studies with radiolabeled iron-59 in K562 and bone marrow cells confirmed the increased uptake and incorporation of iron-59 in the cells and haem using tropolone, maltol and other lipophilic chelators. In contrast, a decrease in the uptake and incorporation of iron-59 in the same cells and haem was observed using mimosine, L1, DF and other hydrophilic chelators [91]. Overall, it appears that lipophilic chelators forming lipophilic chelator iron complexes transfer iron into cells in contrast to hydrophilic and charged chelators which inhibit this process.

Many other cell studies were carried out to identify the influence of phytochelators and synthetic chelators on specific iron metabolic pathways, which were related to other metabolic pathways such as DNA synthesis and diseases such as cancer. The results from such studies provided further information on the efficacy and toxicity of the chelators and their metal complexes. Within this context the cytotoxic and DNA-inhibitory effects of phytochelators and synthetic chelators and their iron complexes was studied and compared with the established anticancer drugs doxorubicin and mitoxantrone in the human leukaemic myeloid K-562, HL60, ML2 and U-937 cell lines. Unlike mimosine, kojic acid, DF and other hydrophilic chelators, tropolone, 8-hydroxyquinoline and other highly lipophilic chelators and especially their lipophilic iron complexes showed comparable cytotoxic and DNA synthesis inhibitory effects similar to the anticancer drugs at low concentrations (0.02 mM) [92].

Similar results of iron transfer, transferrin receptor synthesis and DNA synthesis were obtained in different studies using the K-562 and U-937 cell lines between the hydrophilic and lipophilic chelators and their iron complexes [93]. The lipophilic chelators and especially tropolone suppressed the accumulation of transferrin supplied iron in K-562 and less so in U-937. Similarly, the lipophilic chelators were also shown to be cytotoxic with U-937 cells and the latter to be more sensitive to toxicity than K-562 cells. DNA synthesis was inhibited by lipophilic chelators, which was reversed to a high degree with the addition of iron. Maltol and hydrophilic chelators such as L1 appear to increase DNA synthesis in the same model. Increase in transferrin receptor synthesis was observed in incubations of non-cytotoxic hydrophilic chelators such as L1 and mimosine, suggesting a possible regulatory link between the intracellular low molecular weight iron pool and transferrin receptor expression [93].

The effect on iron uptake and release of the phytochelators mimosine and maltol and of selected L1 analogues and of DF in a range of concentrations (0.2–0.002 mM) were investigated in a model of mouse peritoneal macrophages pulsed with iron-59—transferrin-antitransferrin immune complexes [94]. Mimosine and maltol were more effective than DF, but less effective than L1 and 1-ethyl-3-hydroxy-2-methylpyridin-4-one (L1NEt) in the release of iron from mouse peritoneal macrophages. None of the phytochelators or synthetic chelators increased iron uptake by mouse peritoneal macrophages in comparison to controls suggesting that the chelator iron complexes studied did not recycle iron through the reticuloendothelial system. None of the chelators showed cytotoxic effects in this model [94].

The ability of the phytochelators mimosine and maltol and of selected L1 analogues as well as DF (0.20–0.35 mM) for antibacterial activity or the promotion of growth of the bacterial strains *Y. enterocolitica*, *S. epidermitis*, *E. coli* and *P. aeruginosa* was examined in a number of studies. Maltol inhibited the growth of all bacteria strains and the same results were observed with mimosine with the exception of *Y. enterocolitica* where the growth was promoted. The results of L1 were similar to maltol whereas the iron-nitriloacetic acid (NTA or 2-[bis(carboxymethyl)amino]acetic acid) complex and DF promoted the growth of almost all bacterial strains [95].

In general, phytochelators have variable effects on different cell types in relation to cellular iron metabolic pathways, DNA synthesis, cell survival, antimicrobial, anticancer and other biological activities. These effects depend on several parameters such as the chemical structural characteristics, the lipid/water partition coefficient of the phytochelators and their iron complexes and minimal critical concentration that can be achieved for biological activity. Within this context, in each case maximum biological and pharmacological activity can be achieved by targeting the appropriate metabolic pathways and selecting the phytochelator with the most appropriate properties.

### 7.3. The Selection of the Iron Phytochelators Mimosine, Tropolone and Maltol for the Treatment of Iron Overload and Other Diseases of Iron Imbalance and Toxicity

The α-ketohydroxy metal binding site present in aromatic and heteroaromatic rings was identified as an original, specific and promising chelating site for iron and also the related chelators as possible candidates for development for clinical use [57,58,59]. In particular, the α-ketohydroxy aromatic and heteroaromatic phytochelators such as mimosine, tropolone and maltol are orally active and appear to have high specificity and affinity for iron, are stable at physiological and acidic conditions and form stable iron complexes and also appear to have many other biologically important properties (Table 2) [57,58,59].

Within this context mimosine has been originally tested and compared with the approved chelating drugs DF and L1, in a series of studies of iron mobilisation from the proteins of iron transport and storage including transferrin, lactoferrin, ferritin and haemosiderin [96,97,98,99]. In most studies iron mobilisation by mimosine from these proteins was superior to tropolone and maltol and almost equivalent or slightly lower than L1. In particular, in one study of iron mobilisation from transferrin the rate of iron removal by mimosine was similar to L1 but iron was preferably removed from the N terminal site by comparison to L1 which was from the C terminal site of transferrin [100]. Very few chelating drugs and phytochelators like L1 and mimosine have been shown to mobilise iron from transferrin and lactoferrin effectively suggesting that such chelators have a great potential in iron mobilisation *in vivo*. In contrast DF and DFRA are not effective in the mobilisation of iron from these two proteins under similar conditions [9,10,100].

The encouraging results from the effective iron mobilisation of phytochelators and especially mimosine from the proteins of iron storage and transport prompted further investigations for the characterisation of their iron binding properties. In a series of studies the physicochemical properties of the leading phytochelators mimosine, tropolone and maltol and their red/orange colour iron complexes were examined and characterised at physiological and other conditions. In preliminary *in vitro* studies it has been shown that these three α-ketohydroxy phytochelators form stable iron complexes at physiological pH of 3 (chelator):1 (Fe) stoichiometry. At acidic pH, e.g., stomach conditions, 1 (chelator):1 (Fe) stoichiometry complexes have been identified and between acidic and physiological pH a 2 (chelator):1 (Fe) stoichiometry iron complexes are also formed, similar to the iron complexes stoichiometry formed using L1 [57,58,59,101,102,103].

At physiological pH mimosine and its iron complex are charged and hydrophilic, whereas tropolone and maltol and their iron complexes are neutral and lipophilic (Table 2). The lipophilic chelators forming lipophilic chelator iron complexes appear to transfer iron into red blood cells at a different rate which increases proportionally according to their lipophilicity [90,91]. The lipophilic chelators can also transfer iron into other cell types, e.g., cancer cell lines causing cytotoxic and anticancer effects, which in the case of tropolone was similar to the anticancer drugs doxorubicin and mitoxantrone [92]. Maltol and its iron complex transfer iron across the cell membrane at a slower rate than tropolone. However, unlike tropolone, maltol is not cytotoxic to most cell lines.

In contrast to the lipophilic chelators, the hydrophilic and charged chelating drugs DF and L1 and also the phytochelator mimosine inhibit the transport of iron into cells. It was observed in general that hydrophilic chelators as well as their hydrophilic chelator iron complexes are not cytotoxic by comparison to the lipophilic chelators and their iron complexes [90,91].

In addition to the *in vitro* results many phytochelators including the three phytochelators mimosine, tropolone and maltol and other synthetic chelators were investigated in *in vivo* models of iron metabolic disorders including iron overload and iron deficiency anaemia. In a study of the effect of chelators on iron absorption in mice the iron complexes of the phytochelators mimosine, tropolone and maltol, caffeic acid, 2,3-dihydroxybenzoic acid, 3,4-dihydroxybenzoic acid and citric acid were compared to known chelators such as DF, L1 and DTPA [35]. The lipophilic chelators, including maltol increased iron absorption in repeated experiments with the absorbed iron been stored mainly in the liver and utilised for the production of haemoglobin [35]. In contrast hydrophilic chelators such as DF, DTPA and mimosine caused a substantial reduction in iron absorption. In the same animal model tropolone formed an insoluble iron complex which overall caused a decrease in iron absorption, similar to other insoluble chelator iron complexes [35].

The study of the iron mobilisation effect of phytochelators started with the screening of maltol in the original testing procedure developed for chelators intended for clinical use in thalassaemia [57]. The level of iron excretion in mice caused by maltol in these and other studies that followed was much lower than that caused by L1 and DF and as a result maltol’s development for possible clinical application in iron overload was not pursued any further [57]. In contrast, orally administered tropolone was more effective in increasing iron excretion than L1 and DF at similar doses in the same mouse model, but it also appeared to cause neurotoxic effects and especially convulsions in mice. Tropolone caused equivalent iron excretion and was much less toxic when used at a ten times lower oral dose (20 mg/kg) than L1 and DF (200 mg/kg) in the same model (unpublished) [36].

The *in vitro* results of iron mobilisation by mimosine and especially the removal of iron from transferrin, similar to L1 were encouraging for further development and application in iron overloading diseases. However, the toxicity reported in animals feeding on plants containing mimosine were not encouraging, despite that the major toxic agent was 3,4-dihydroxypyridine, a breakdown metabolic product of mimosine [101,102]. In a preliminary study in iron overloaded rabbits the amount of iron excreted by mimosine in the urine was equivalent to that caused by L1 at the same dose (unpublished) [36]. Further evidence of iron removal from animals has been previously noted by other investigators studying the effect of mimosine on iron and other metal ion excretion [103,104]. These preliminary results were very encouraging but further development of mimosine in the treatment of iron overload was delayed because of the lack of commercial interest and also because of the increasing academic and commercial interest in the development of L1.

### 7.4. Clinical Investigations and Human Use of Mimosine, Tropolone, Maltol and Other Iron Phytochelators

The prospects of clinical investigations and use of the then three leading phytochelators mimosine, tropolone and maltol increased following the preclinical *in vitro* and animal testing in different models related to iron metabolism and toxicity. Within this context, different trends are developing for the possible clinical application of the three phytochelators. Some information on the interaction of mimosine, tropolone and maltol with humans was previously available since the three phytochelators are naturally occurring plant products which have been used or were in contact with humans for hundreds of years.

Maltol is a naturally occurring compound found in the bark of larch tree, in pine needles, in roasted malt and in bread. It is also formed during caramelization and has been widely used and marketed for many years as a food additive and as a flavour enhancer. Maltol was first identified as a potential chelator for increasing iron absorption in the original red blood cell and rat jejunum permeation screening studies of phytochelators and of synthetic chelators which were screened for potential clinical use in iron overload, iron deficiency and other diseases of iron metabolism [57]. In the rat jejunum permeation screening studies the chelator was used at a six molar ratio excess over iron (0.1 mM). In these studies maltol was identified to be more effective than the other phytochelators catechol and citrate for use in the treatment of iron deficiency anaemia [57].

Further development of the maltol iron complex in animal and clinical studies continued for many years and is still ongoing for different categories of iron deficiency anaemia conditions including patients with inflammatory bowel disease at phase III clinical trials [105,106,107]. In the latter case, the ferric maltol administration (30 mg, twice daily) for up to 12 weeks increased haemoglobin levels in two thirds of the patients. No toxic side effects were reported, suggesting that ferric maltol can be used as an alternative to intravenous iron in iron deficiency anaemia patients with inflammatory bowel disease [107].

Mimosine or leucenol, is a non-protein amino acid which is found in the herb *Mimosa pudica* and other mimosa species and also the related legume leucaena, which is found as 16 different species and over 800 varieties. The leaves and seeds of leucaena have been used and consumed as a food product by millions of people in Central America, India, Thailand, Philippines, Indonesia and many other South East Asia countries for hundreds of years.

Despite the fact that many toxic side effects have been reported in animals feeding on leucaena leaves and seeds, no similar toxicity has been reported in humans. In animals the toxic side effects include alopecia, anorexia, weight loss, excessive salivation, eosophageal lesions, enlarged thyroid and low circulating concentrations of thyroid hormones. Mimosine is a known antimitotic and depilatory agent in animals but the main toxicity is exerted by the mimosine metabolite 3,4-dihydroxypyridine which is a potent goitrogen [101]. It’s other metabolite in animals is 2,3-dihydroxypyridine, which is less toxic. All the toxic side effects reported in animals feeding on plant products containing mimosine appear to be reversible on switching to alternative food products which do not contain mimosine.

Despite the promising *in vitro* iron mobilising and *in vivo* iron removal effects of mimosine, no clinical studies have been reported in iron-loaded patients. In preliminary clinical studies of powder prepared from seeds of leuceana using different doses of up to 5 g twice daily, the seed preparation was well tolerated and cause no apparent toxicity.

The inhibition of iron absorption in animals or the inhibition of transfer of iron in many cell types caused by mimosine at levels similar to those caused by DF suggest that mimosine may have possible clinical application in acute iron poisoning. Mimosine has many other biological effects and the main other research interests for clinical application are the antioxidant and anticancer activity including the inhibition of the key iron containing enzyme ribonucleotide reductase involved in DNA synthesis [108,109].

The distribution of the plants containing mimosine is mainly in developing countries of South East Asia where thalassaemia is endemic. Since many plant products containing mimosine are consumed by humans the possibility of clinical development and application in the treatment of iron overload in thalassaemia and other conditions should be further investigated. Such efforts will be particularly helpful for patients who are not receiving any chelation treatment due to the high cost or idiosyncratic toxicity related to treatments by the chelating drugs L1, DF and DFRA [7].

Tropolone is a natural product found mainly in plants like the Western Red Cedar and shown to have many biological activities, including iron chelation properties and the ability to mobilise iron from transferrin [56,92,110]. There is no information on clinical investigations or human use of tropolone as a food product. Similar to other phytochelators, considerations of risk/benefit assessment could also apply to tropolone or naturally occurring biologically active homologues of tropolone like hinokitiol and purpurogallin which have shown iron chelating, antioxidant and other biological activities [111,112,113,114].

Recent clinical trials of the flavonoid phytochelator silybin or extracts containing silybin were reported in patients with hereditary haemochromatosis and thalassaemia with encouraging results (Figure 10) [115,116,117]. In the latter silybin was administered in combination with DFRA and the overall therapeutic effects were limited and not sufficient for achieving negative iron balance [117]. It is envisaged that combination therapies of phytochelators with chelating drugs as shown with silybin and DFRA and ascorbic acid with DF may improve the efficacy of the overall chelation therapy in comparison to monotherapies [44,117].

The clinical development of phytochelators such as tannins and phytates which inhibit iron absorption, including clinical investigations for the possible treatment of hereditary haemochromatosis, acute iron poisoning and colorectal cancer should further be considered [60,118,119]. In contrast, the use of maltol, ascorbic acid and related chelators which are known to increase iron absorption should further be considered for development in the treatment of iron deficiency anaemia [43,105,106,107]. Similar effects on increased iron absorption have also been shown in animals by 8-hydroxyquinoline which is not only a synthetic chelator but appears to occur naturally in some plant species and have a wide range of biological activities and uses in clinical diagnostics involving other metals in addition to iron (Figure 10) [17,120,121,122,123,124,125].

The detoxification of other toxic metals by phytochelators such as aluminium, which has been previously shown in renal dialysis patients using L1 and DF, needs further investigation and development [25,30]. The ability of mimosine to mobilise iron from transferrin may also be promising for aluminium detoxification, since the α-ketohydroxy metal binding site appears to have high affinity for both metal ions and furthermore the aluminium properties *in vivo,* such as transport in plasma by transferrin is similar to iron [25,30,100,126]. Many other possible clinical applications are anticipated by phytochelators of different metal chelating sites similar to those described by the chelating drugs L1, DF and EDTA [9,10,16,24,127]. Within this context, the interaction of phytochelators with metallothioneins, which are able to bind a variety of metal ions by the formation of mercaptide bonds between the numerous cysteine residues present in the proteins and the metal ions are considered important in heavy metal detoxification in plants and animals [128,129].

## 8. Conclusions

Many phytochelators including polyphenols and also other naturally occurring plant products with specific metal binding sites or structures need to be identified and characterised for their iron and other metal binding properties *in vitro* and *in vivo*. Selected phytochelators could be developed and introduced for clinical use in the treatment of iron overload and other diseases of iron metabolism and toxicity, as well as many other conditions which have been identified during the development of L1, mimosine, maltol, tropolone and other chelators [9,10,57].

Despite that the major emphasis in the development of polyphenols is in relation to their free radical quenching antioxidant properties, many of the polyphenolic phytochelators have the additional advantage of preventing the iron and copper catalyzed formation of free radicals and free radical cascades by binding these metal ions and rendering them redox inactive. This double effect in the antioxidant activity by polyphenolic phytochelators can be proved more effective in pathological conditions related to free radical damage and also in ageing.

In all cases of possible clinical application of an investigational new drug (IND) a risk/benefit assessment is required, as well as comparison with existing therapies. Such comparison can extend to parameters related to efficacy, toxicity and availability for each individual patient. This approach is based on personalised medicine and ADMET considerations which are the current trend for the development of optimal therapies in individual patients [9].

The possibility of the use of phytochelators in combination with other chelating or other drugs in many other diseases is another option which has to be considered for further development. Such combinations also require a risk/benefit assessment in comparison to existing therapies and also the assessment of the beneficial effects for each individual patient [130].

The major drawbacks in the clinical development of phytochelators and other plant products are mainly the lack of *in vivo* and clinical data which are the evidence based medicine considerations for therapeutic use. It appears that in general the vast commercial area of neutraceuticals is based on *in vitro* and insufficient or anecdotal clinical findings. Within this context therapeutic claims should be based on many parameters such as therapeutic window of minimum/maximum effectiveness, identification of non-toxic dose ranges, bioavailability/ADMET profile, antioxidant/prooxidant effects *etc.* [131,132,133].

The introduction of new drugs including neutraceuticals is usually based on commercial and not ethical considerations [7]. This approach affects many patients who have no sufficient financial resources for buying expensive drugs or efficient health care [7,134]. The major advantage of the utilisation of natural plant products is that these are easily available from natural resources and can be used as main or alternative or adjuvant therapies in many conditions, including iron overload in thalassaemia and iron deficiency anaemia which are prevalent in developing countries.

The development of many classes of phytochelators is a challenging and unexplored area which needs further investigation for the development of new pharmaceutical entities which can be utilised in many diseases in addition to iron overload and iron deficiency, such as cancer, neurodegenerative, cardiovascular, renal and infectious diseases and also in ageing [2,135,136,137,138,139,140]. The treatment of Friedreich ataxia and other neurodegenerative diseases using chelators is particularly promising at the moment, especially since no other treatments appear to be effective [2,9,32]. Further investigations are also needed in relation to the mechanisms and prevention of toxicity associated with phytochelators and their iron complexes. For example the redox properties of iron complexes of different stoichiometry could be potentially toxic, similar to the toxicity of the 1L1:1Fe and 2L1:Fe stoichiometry complexes suggested for L1, as well as for other complexes such as the 1EDTA:1Fe complex [41,141].
